# *Salmonella* Typhi, Paratyphi A, Enteritidis and Typhimurium core proteomes reveal differentially expressed proteins linked to the cell surface and pathogenicity

**DOI:** 10.1371/journal.pntd.0007416

**Published:** 2019-05-24

**Authors:** Sara Saleh, Sandra Van Puyvelde, An Staes, Evy Timmerman, Barbara Barbé, Jan Jacobs, Kris Gevaert, Stijn Deborggraeve

**Affiliations:** 1 Department of Biomedical Sciences, Institute of Tropical Medicine, Antwerp, Belgium; 2 VIB Center for Medical Biotechnology, Ghent, Belgium; 3 Department of Biomolecular Medicine, Ghent University, Ghent, Belgium; 4 Department of Clinical Sciences, Institute of Tropical Medicine, Antwerp, Belgium; 5 Department of Microbiology and Immunology, KU Leuven, Leuven, Belgium; University of Colorado Health Sciences Center, UNITED STATES

## Abstract

**Background:**

*Salmonella enterica* subsp. *enterica* contains more than 2,600 serovars of which four are of major medical relevance for humans. While the typhoidal serovars (Typhi and Paratyphi A) are human-restricted and cause enteric fever, non-typhoidal *Salmonella* serovars (Typhimurium and Enteritidis) have a broad host range and predominantly cause gastroenteritis.

**Methodology/Principle findings:**

We compared the core proteomes of *Salmonella* Typhi, Paratyphi A, Typhimurium and Enteritidis using contemporary proteomics. For each serovar, five clinical isolates (covering different geographical origins) and one reference strain were grown in vitro to the exponential phase. Levels of orthologous proteins quantified in all four serovars and within the typhoidal and non-typhoidal groups were compared and subjected to gene ontology term enrichment and inferred regulatory interactions. Differential expression of the core proteomes of the typhoidal serovars appears mainly related to cell surface components and, for the non-typhoidal serovars, to pathogenicity.

**Conclusions/Significance:**

Our comparative proteome analysis indicated differences in the expression of surface proteins between *Salmonella* Typhi and Paratyphi A, and in pathogenesis-related proteins between Salmonella Typhimurium and Enteritidis. Our findings may guide future development of novel diagnostics and vaccines, as well as understanding of disease progression.

## Author summary

With an estimated 20 million typhoid cases and an even higher number of non-typhoid cases the health burden caused by salmonellosis is huge. Salmonellosis is caused by the bacterial species *Salmonella* enterica and over 2500 different serovars exist, of which four are of major medical relevance for humans: Typhi and Paratyphi A cause typhoid fever while Typhimurium and Enteritidis are the dominant cause of non-typhoidal *Salmonella* infections. The proteome is the entire set of proteins that is expressed by a genome and the core proteome are all orthologous proteins detected in a given sample set. In this study we have investigated differential expression of the core proteomes of the *Salmonella* serovars Typhi, Paratyphi A, Typhimurium and Enteritidis, as well as the regulating molecules. Our comparative proteome analysis indicated differences in the expression of surface proteins between the serovars Typhi and Paratyphi A, and in pathogenesis-related proteins between Typhimurium and Enteritidis. Our findings in proteome-wide expression may guide the development of novel diagnostics and vaccines for *Salmonella*, as well as understanding of disease.

## Introduction

The gram-negative bacterial genus *Salmonella* is divided in two species, *Salmonella enterica* and *Salmonella bongori*. Only the *Salmonella enterica* subspecies *enterica* is of clinical relevance for humans and is further classified into more than 2,600 serovars. The human restricted serovar Typhi (STY) and the closely related serovar Paratyphi A (SPTA) cause enteric fever [1], while the generalist serovars Typhimurium (STM) and Enteritidis (SENT) are the most important causes of non-typhoidal salmonellosis [2]. Enteric fever is a systemic disease that affects more than 27 million people worldwide and leads to more than 200,000 deaths annually [3,4]. While STY and SPTA both cause a systemic disease, SPTA causes a milder disease with a shorter incubation time [5]. In the last 20 years, the number of infections with SPTA has significantly increased in Asia [6]. The global burden of non-typhoidal *Salmonella*, a common cause of food poisoning that is usually characterized by localized gastroenteritis, is even higher with an estimated 93.8 million cases and 155,000 deaths each year [2]. Moreover, invasive non-typhoidal *Salmonella* has emerged as an important cause of bloodstream infection in Sub-Saharan Africa in both adults and children, and the incidence of invasive non-typhoidal *Salmonella* is estimated at 3.4 million cases with more than 600,000 deaths each year [7].

Comparative genomics of *Salmonella enterica* has revealed specific genetic fingerprints associated with invasive disease and host adaptation [8,9]. A comparative analysis of 8 typhoidal and 27 non-typhoidal *Salmonella* genomes demonstrated presence of typhoid-specific protein families which include virulence factors such as Vi polysaccharide pilus related proteins [10]. In addition, an *in silico* comparative analysis of *Salmonella* genomes identified 469 genes involved in the central anaerobic metabolism which was intact in gastrointestinal pathogens (SENT and STM among others) but decaying in extra-intestinal pathogens, such as STY and SPTA. This metabolic advantage might have a role in competing with other bacteria in the inflamed gut, thereby enhancing transmission of the gastrointestinal pathogens [11]. However, not all phenotypic differences in typhoidal and non-typhoidal *Salmonella* can be explained by presence or absence of functional genes. Investigating differential expression of the core proteomes (defined as all orthologous proteins quantified in a given sample set) between *Salmonella* serovars [12], and the regulating molecules involved, can reveal additional insights in the adaptations to different host environments and pathogenesis, as well as reveal the expression of potential vaccine and diagnostic targets.

In the last decade, mass spectrometry (MS) based proteomics has advanced rapidly and provides a comprehensive view on the proteins that are expressed by an organism. In clinical microbiology laboratories, MALDI-TOF MS is routinely used for bacterial genus and species identification [13]. In research, proteomics was used to characterize the proteomes of *Salmonella* Typhimurium and Enteritidis under specific *in vitro* culture conditions mimicking the phagosome [14,15], to identify proteins that were expressed by *Salmonella* Typhimurium isolated from infected macrophages [16], and to study antimicrobial resistance and virulence in *Salmonella* Typhimurium [17–19]. Next to proteome analysis within single serovars, comparative proteome studies have been conducted to assess the proteome variability between different *Salmonella* serovars. However, these studies used laboratory reference strains which may not represent the currently circulating clinical strains [20–22].

Here, we conducted a comparative analysis of the core proteomes of the clinically most relevant *Salmonella enterica* serovars: Typhi, Paratyphi A, Typhimurium and Enteritidis, using 20 *Salmonella* strains isolated from patients covering various geographical origins, as well as one reference strain per serovar. Our findings show that differential expression of the core proteome of the typhoidal serovars is mainly related to cell surface components and, for the non-typhoidal serovars, to pathogenicity.

## Methods

### Bacterial strains and growth conditions

Five clinical isolates per *Salmonella* serovar Typhi, Paratyphi A, Typhimurium and Enteritidis were selected from the strain collection at the clinical laboratory of the travel clinic of the Institute of Tropical Medicine, Antwerp, Belgium for shotgun proteome analysis. One ATCC reference strain for each *Salmonella* serovar was added to the sample set and for the *Salmonella* Typhi reference strain, a clinical strain was certified (Table 1). Given that the burden of typhoid fever and invasive non-typhoidal salmonellosis is highest in Asia and Africa respectively, we have selected representative strains from different countries covering both continents. All *in vitro* incubation was done at 37°C. Minimum and maximum temperatures were recorded and ranged between 35°C and 37°C. As all clinical strains have been isolated from patients, the strains were revived from Microbank cryogenic vials (Pro-Lab Diagnostics) on blood agar (BD Columbia Agar, 5% sheep blood) and grown overnight at 37°C. Single colonies were sub-cultured on MacConkey agar (BD MacConkey II Agar) and grown overnight at 37°C. Colonies were further solubilized into 3 ml of synthetic growth medium and supplemented with 1% glucose (Teknova HI-DEF Azure Media) until the OD was 0.06, and 250 μl of this suspension was inoculated into 5 ml of synthetic medium supplemented with 1% glucose and grown at 37°C with shaking at 220 rpm until mid-log phase (OD 0.5-OD 0.6). The Teknova HI-DEF Azure synthetic medium (S1 File) is based on the medium described by Neidhardt et al. [23].

**Table 1:** Geographical origin and year of isolation of the *Salmonella enterica* Typhi, Paratyphi A, Typhimurium and Enteritidis strains.

ID strain	*Salmonella enterica* serovar	Geographic origin	Year of isolation
**Clinical isolates**			
9092306	Typhi	Bangladesh	2009
9121199	Typhi	Burkina Faso	2009
2427†	Typhi	Cambodia	2010
3182/3†	Typhi	DRC*	2010
12091815	Typhi	Thailand	2012
8041131	Paratyphi A	India	2008
8121108	Paratyphi A	Senegal	2008
1964†	Paratyphi A	Cambodia	2010
12082646	Paratyphi A	India	2012
12122069	Paratyphi A	Myanmar	2012
3011187	Typhimurium	Ethiopia	2003
2371	Typhimurium	Cambodia	2010
11082746	Typhimurium	Malawi	2011
HRG039VD28	Typhimurium	The Gambia	2013
11185/3†	Typhimurium	DRC*	2014
9001877	Enteritidis	Cambodia	2009
3252/3†	Enteritidis	DRC*	2010
10080748	Enteritidis	Nigeria	2010
12050236	Enteritidis	Senegal	2012
12080487	Enteritidis	Indonesia	2012
**Reference isolates**			
ITM00032304‡	Typhi	Senegal	2000
ATCC9150	Paratyphi A	Malaysia	1993
ATCC14028	Typhimurium	unknown	1960#
ATCC13076	Enteritidis	unknown	unknown

* Democratic Republic of the Congo

# ATCC 14028 is a descendant of CDC 60–6516, which is a strain isolated in 1960 from pools of hearts and livers of 4-week-old chickens.

†Obtained from microbiological surveillance studies in the respective countries. The other strains were obtained from patients at the travel clinic of ITM.

‡Clinical strain certified by the Belgian National Reference Centre for *Salmonella* and *Shigella* (ISP-WIV, currently Sciensano, Brussels).

### Protein extraction and in-solution digestion

Upon harvesting the bacteria, duplicate samples of 1 ml were taken from each culture and centrifuged at 5000 x g for 10 min at 4°C and the cell pellets were washed twice with phosphate buffered saline (PBS). Duplicate samples are thus further considered as technical replicates. Proteins were extracted from the bacterial pellets with the Qproteome Bacterial Protein Prep Kit (Qiagen) following the manufacturer’s instructions. Briefly, after snap-freezing on dry ice, bacterial cell pellets were thawed on ice for 15 minutes. Cell pellets were re-suspended 750 μl of lysis buffer supplemented with lysozyme and Benzonase Nuclease, all included in the extraction kit. EDTA-free protease inhibitor (Roche) was added to a final concentration of 2%. After incubation on ice for 30 minutes, lysates were centrifuged at 14,000 for 30 minutes to pellet the cellular debris, and the supernatant was collected. The protein concentration was determined with the BCA Protein Assay Kit (Pierce) (S1 Table). Proteins were reduced with 15 mM tris(2-carboxyethyl)phosphine hydrochloride (TCEP-HCl) and alkylated with 30 mM iodoacetamide (IAM) for 15 min in the dark while shaking at 37°C. The buffer was exchanged to digestion buffer (50 mM ammonium bicarbonate, pH 7.9) using G-25 illustra NAP-5 gel filtration columns (GE Healthcare). The eluates were then heated at 99°C for 5 min, put immediately on ice and, after cooling, sequencing grade modified trypsin (Promega) was added to a 1:100 trypsin to protein ratio upon which digestion proceeded at 37°C for 16 h. The trypsin activity was stopped by adding 60 μl of 10% trifluoroacetic acid (TFA) (0.6% final concentration).

### LC-MS/MS analysis

The peptide mixtures were subjected to LC−MS/MS analysis using an Ultimate 3000 RSLC nano LC (Thermo Scientific, Bremen, Germany) in-line connected to a Q Exactive mass spectrometer (Thermo Fisher Scientific). The sample mixture was first loaded on a trapping column (made in-house, 100 μm internal diameter (I.D.), 20 mm long, filled with 5 μm C18 Reprosil-HD beads, Dr. Maisch, Ammerbuch-Entringen, Germany). After flushing from the trapping column, the peptides were loaded on an analytical column (75 μm I.D., 400 mm long and filled with 3 μm C18 Reprosil-HD beads (Dr. Maisch)) packed in the needle PicoFrit SELF/P PicoTip emitter (PF360-75-15-N-5 (NewObjective, Woburn, USA)). Peptides were loaded with loading solvent (0.1% TFA in water) and separated with a linear gradient from 98% solvent A’ (0.1% formic acid in water) to 40% solvent B′ (0.1% formic acid in water/acetonitrile, 20/80 (v/v)) in 130 min at a flow rate of 300 nL/min. This was followed by a 15 min wash reaching 99% solvent B’. The mass spectrometer was operated in data-dependent, positive ionization mode, automatically switching between MS and MS/MS acquisition for the 10 most abundant peaks in a given MS spectrum. The source voltage was 3.4 kV and the capillary temperature was at 275°C. One MS1 scan (m/z 400−2000, AGC target 3 × 10^6^ ions, maximum ion injection time 80 ms) acquired at a resolution of 70,000 (at 200 m/z) was followed by up to 10 tandem MS scans (resolution 17,500 at 200 m/z) of the most intense ions fulfilling the defined selection criteria (AGC target 5 × 10^4^ ions, maximum ion injection time 60 ms, isolation window 2 Da, fixed first mass 140 m/z, spectrum data type: centroid, underfill ratio 2%, intensity threshold 1.7xE^4^, exclusion of unassigned 1, 5–8, >8 charged precursors, peptide match preferred, exclude isotopes: on, dynamic exclusion time 20 s). The HCD collision energy was set to 25% normalized collision energy and the polydimethylcyclosiloxane background ion at 445.120025 Da was used for internal calibration (lock mass). The mass spectrometry proteomics data have been deposited to the PRIDE Archive (http://www.ebi.ac.uk/pride/archive/) via the PRIDE partner repository with the data set identifier PXD011154 (username: reviewer00797@ebi.ac.uk; password: hN5SqXtY).

### MS data processing

Raw MS files were analyzed by MaxQuant [24] version 1.5.0.25 and MS/MS spectra were searched against the translated protein sequences of the annotated genomes of *Salmonella* Typhi CT18 (NCBI accession number AL513382.1) [25], Paratyphi A ATCC 9150 (CP000026.1) [26], Typhimurium 14028S (CP001363.1) [27], and Enteritidis PT4/P125109 (AM933172.1) [28]. The following parameters were applied for the database search: enzyme specificity was set to trypsin/P allowing for a maximum of two missed cleavages; carbamidomethylation of cysteine was set as a fixed modification; methionine oxidation, N-terminal formylation on the protein level and conversion of N-terminal glutamine to pyroglutamate were set as variable modifications. The first search for precursor ions was performed with a mass tolerance of 20 ppm for calibration, while 6 ppm was applied for the main search. For protein identification, at least two unique peptides were required per protein group and the minimum peptide length was set to 7. The false discovery rate for peptide and protein identification was set to 1%. The minimum score threshold for both modified and unmodified peptides was set to 30. MS runs were analyzed with the “match between runs” option between samples of a given serovar. For matching, a retention time window of 42 s was selected. Protein quantification was based on the MaxQuant label-free (MaxLFQ) algorithm. For all other parameters, default settings were applied as advised by the developers.

### Comparative analysis of core proteomes

The MaxQuant output file “proteinGroups.txt” was loaded into Perseus 1.5.0.8. The protein entries were filtered to remove potential contaminants, reverse hits and proteins only identified by site. Then, the LFQ intensities were log2 transformed and data were filtered for proteins containing a minimum number of valid values in 9 out of 12 samples. The log2 transformed data were then normalized by subtracting the median per sample within the dataset. To compare the different *Salmonella* serovars we used orthology mapping. Orthologous genes within the four serovars were retrieved from the Orthologous Matrix (OMA) database [29] with NCBI Taxonomy IDs 220341 (STY), 295319 (SPTA), 550537 (SENT) and 588858 (STM). Statistical significant differences in LFQ intensities were assessed using a two-sided t-test with Bonferroni adjusted *P* values using R. Proteins were considered differentially expressed if they showed a minimal 2-fold change in their overall levels with an adjusted *P*-value lower than 0.05. Principal component analysis (PCA) was done in Perseus 1.5.0.8 using default settings as advised by the developers.

### Functional enrichment analysis

Differentially expressed proteins were subjected to gene ontology (GO) term enrichment to investigate biological processes, molecular function and cellular compartment using the Database for Annotation, Visualization and Integrated Discovery (DAVID) bioinformatics resources 6.7 [30]. Briefly, we have uploaded the differentially expressed core proteins as an input list and performed GO term enrichment analysis against a background list with default settings (count threshold is 2 and EASE threshold is 0.1).

### Regulatory network analysis

To infer regulatory interactions that can explain differential expression profiles we used the PheNetic web server (http://bioinformatics.intec.ugent.be/phenetic/#/index) with default settings (Cost is 0.1, Pathlength is 4 and k-best paths is 20) and upstream run mode [31]. Input data consisted of the available interaction network for *Salmonella* Typhimurium LT2 (http://bioinformatics.intec.ugent.be/phenetic/index.html#/network), the list of detected proteins that are shared by two groups, and the list of differentially expressed proteins with *P*<0.05.

### Ethics statement

The clinical *Salmonella* isolates were obtained through the project “Surveillance of antimicrobial resistance among consecutive blood culture isolates in tropical settings”, within the Third Framework Agreement between the Belgian Directorate of Development Cooperation (DGD) and the Institute of Tropical Medicine (ITM), Antwerp, Belgium. The partner institutes involved in this surveillance project that provided strains were: Sihanouk Hospital Centre of Hope, Phnom Penh, Cambodia and Institut National de Recherche Biomédicale, Kinshasa, Democratic Republic of the Congo. Ethical approval for the Microbiological Surveillance was granted by the Institutional Review Board at the ITM in Antwerp, by the Ethics Committees of the Antwerp University (Belgium). Ethical approval for the Microbiological Surveillance Study was granted by the Institutional Review Board of ITM, the Ethics Committee of Antwerp University and the competent ethical committees from the DR Congo and Cambodia respectively. The *Salmonella* Typhimurium isolate from The Gambia was received from the Medical Research Council (MRC) Keneba, MRC The Gambia. Ethical approval was granted by the Gambia Government/MRC Joint Ethics Committee. The remaining strains were obtained from patients presenting at the travel clinic at ITM, ethical approval was granted by the Institutional Review Board of ITM. All isolates and subsequent biological samples were anonymized.

## Results

### *Salmonella* proteins identified by LC-MS/MS

The reference genomes of STY, SPTA, SENT and STM used in our analysis contain 4,600, 4,095, 4,318 and 5,372 protein-encoding genes, respectively. In total, 3596 orthologous genes in the four serovars were retrieved from the OMA database and 1,414, 1,558, 1,222 and 1,099 proteins were detected by LC-MS/MS analysis in the STY, SPTA, SENT and STM strains, respectively. Protein detection in technical replicates showed Pearson correlation coefficients higher than 0.92 for all samples, except for the STM strain from Ethiopia with a Pearson correlation of 0.86 (S2 Table). Intra-serovar PCA of the LFQ intensities of expressed proteins show little variation in expression levels between strains within the same serovar (S2 File). However, in order to conduct reliable intra-serovar comparisons, more strains should have been included per serovar.

**Fig 1:**
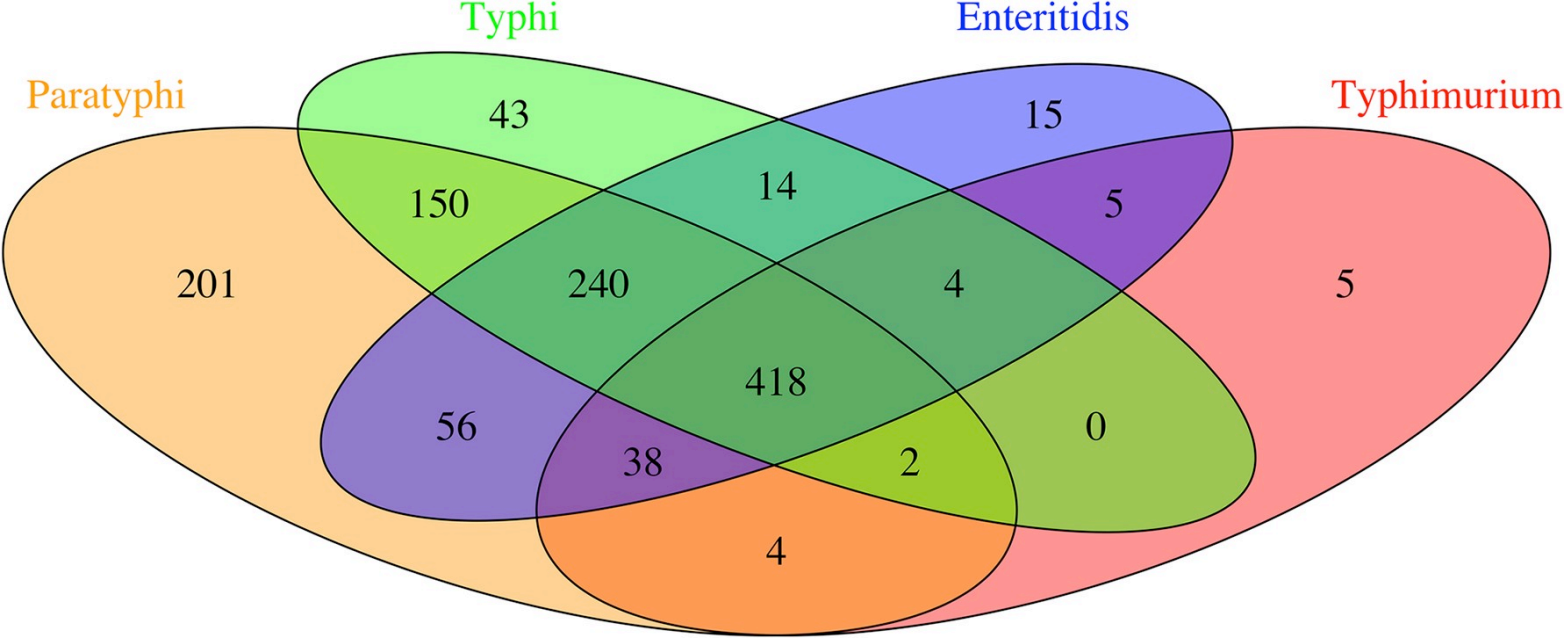
Venn diagram of the orthologous proteins detected by LC-MS/MS in 6 *Salmonella* Typhi, 6 *Salmonella* Paratyphi A, 6 *Salmonella* Enteritidis and 6 *Salmonella* Typhimurium strains.

**Fig 2:**
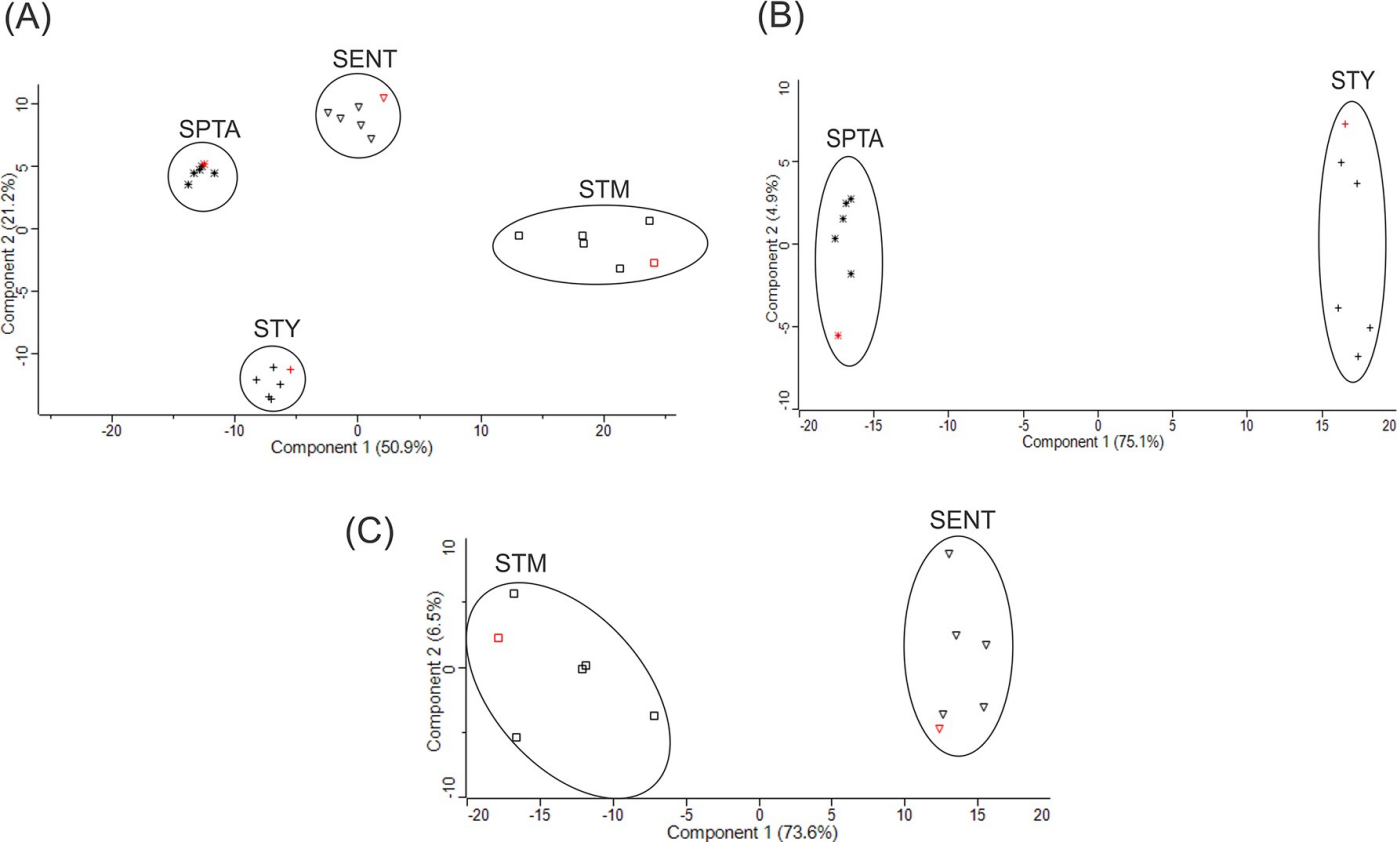
Principal component analysis (PCA) separate serovars based on LFQ intensities. The PCA plots show that the first and second principle components capture ~72% of the variability among the *Salmonella* serovars Typhi (STY), Paratyphi A (STPA), Typhimurium (STM) and Enteritidis (SENT) **(A)**, 80% of the variability between the serovars STY and SPTA **(B)**, and ~80% of the variability between the serovars STM and SENT **(C)**. Reference strains for each serovar are presented in red.

In total, 418 orthologous proteins were detected in all serovars (Fig 1) and expression levels in the typhoidal (STY and SPTA) and non-typhoidal (STM and SENT) *Salmonella* serovars were compared by PCA of the LFQ intensities (Fig 2A). The first two components capture ~72% of the variability in the dataset and show that the typhoidal serovars do not separate from the non-typhoidal serovars based on the observed variability in LFQ intensities. When we compared the typhoidal with the non-typhoidal *Salmonella* strains, a total of 128 proteins showed a minimal 2-fold change in their overall levels with an adjusted *P*-value lower than 0.05 (S3 Table). GO term enrichment of these 128 proteins showed that all GO terms with a *P* value lower than 0.05 are related to translation and structural components of the ribosomes (Table 2).

**Table 2:** Gene ontology functional enrichment analysis of differentially expressed core proteins between typhoidal and non-typhoidal, Typhi and Paratyphi A, Enteritidis and Typhimurium *Salmonella*.

Category	Term	Count	%	*P-value*
**Typhoidal versus non-typhoidal**				
GOTERM_MF_FAT	GO:0005198~structural molecule activity	38	29.46	2.56E-10
GOTERM_MF_FAT	GO:0003735~structural constituent of ribosome	37	28.68	3.13E-10
GOTERM_BP_FAT	GO:0006412~translation	42	32.56	5.99E-07
GOTERM_CC_FAT	GO:0005840~ribosome	37	28.68	9.52E-06
GOTERM_CC_FAT	GO:0030529~ribonucleoprotein complex	37	28.68	2.17E-05
GOTERM_MF_FAT	GO:0003723~RNA binding	29	22.48	2.39E-05
GOTERM_CC_FAT	GO:0043232~intracellular non-membrane-bounded organelle	38	29.46	9.00E-05
GOTERM_CC_FAT	GO:0043228~non-membrane-bounded organelle	38	29.46	9.00E-05
GOTERM_MF_FAT	GO:0019843~rRNA binding	21	16.28	2.82E-04
GOTERM_CC_FAT	GO:0033279~ribosomal subunit	12	9.3	0.024948
GOTERM_MF_FAT	GO:0000049~tRNA binding	7	5.42	0.048571
**STY versus SPTA**				
GOTERM_BP_FAT	GO:0016051~carbohydrate biosynthetic process	10	4.4	5.55E-04
GOTERM_BP_FAT	GO:0008610~lipid biosynthetic process	10	4.4	0.001
GOTERM_BP_FAT	GO:0034637~cellular carbohydrate biosynthetic process	8	3.52	0.006
GOTERM_BP_FAT	GO:0000271~polysaccharide biosynthetic process	7	3.08	0.009
GOTERM_BP_FAT	GO:0009103~lipopolysaccharide biosynthetic process	6	2.64	0.014
GOTERM_BP_FAT	GO:0008653~lipopolysaccharide metabolic process	6	2.64	0.014
GOTERM_BP_FAT	GO:0005976~polysaccharide metabolic process	7	3.08	0.016
GOTERM_BP_FAT	GO:0044264~cellular polysaccharide metabolic process	6	2.64	0.026
GOTERM_BP_FAT	GO:0033692~cellular polysaccharide biosynthetic process	6	2.64	0.026
GOTERM_CC_FAT	GO:0030312~external encapsulating structure	7	3.08	0.033
**STM versus SENT**				
GOTERM_CC_FAT	GO:0019861~flagellum	8	4.14	0.037
GOTERM_CC_FAT	GO:0042995~cell projection	8	4.14	0.037
GOTERM_BP_FAT	GO:0009405~pathogenesis	9	4.66	0.044

### Differentially expressed proteins in *Salmonella* Typhi (STY) and Paratyphi A (SPTA) are associated with the cell surface

**Fig 3:**
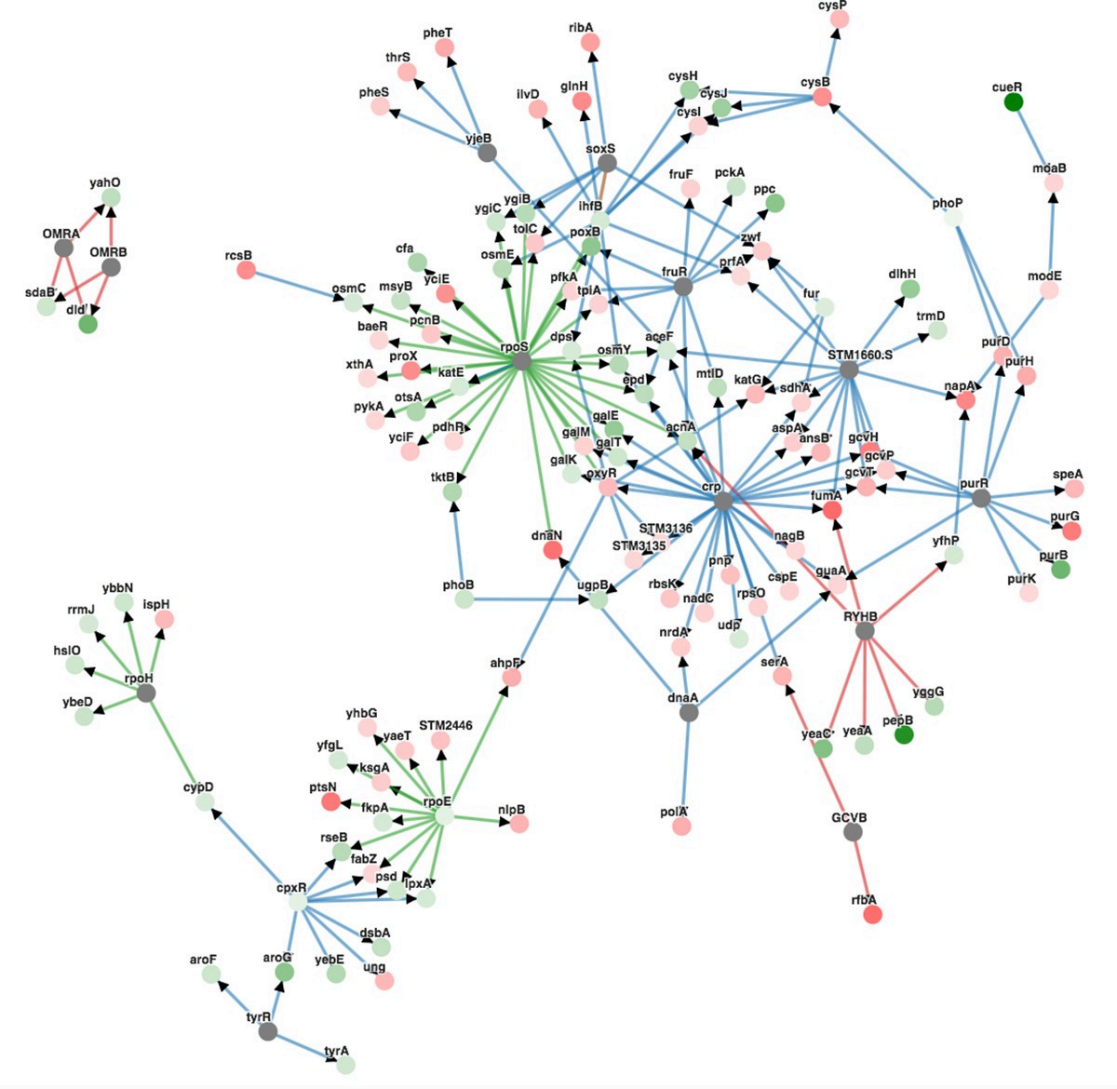
Phenetic sub-network inference analysis of differential protein expression in STY versus SPTA. 122 out of 230 differentially expressed proteins are shown in this sub-network. Red nodes represent proteins with higher expression in SPTA versus STY. Green nodes represent proteins with higher expression in STY versus SPTA. The more intense the color, the higher the level of differential expression. Gray nodes have no differential expression. The color of the edge indicates the interaction type with blue referring to metabolic, green to protein-protein and red to protein-DNA interactions.

A set of 810 core proteins were detected in Typhi and Paratyphi A and their LFQ intensities were used as input for PCA (Fig 2B). The first two components allow a clear separation of the STY from the SPTA strains, covering 80% of the total variation in expression levels. In addition, the PCA shows that clinical isolates do not separate from the reference strains in both serovars. A total of 230 proteins with a minimal 2-fold change in their overall levels and an adjusted *P*-value lower than 0.05 were considered significantly differentially expressed between STY and SPTA strains (S4 Table). GO functional enrichment analysis of these proteins indicated an enrichment of biological pathways that are related to carbohydrate and polysaccharide biosynthesis and metabolism, as well as the external encapsulating structure (Table 2). We have plotted our differential expression data set on the wide interaction network for *Salmonella* Typhimurium LT2. Using the upstream run mode, PheNetic searches for regulatory mechanisms that can explain our observed data set. The inferred sub-network (Fig 3) shows that many differentially expressed proteins are connected to each other by outer membrane, stress and carbohydrate metabolism regulatory proteins such as CpxR, YjeB and CRP, which are not necessarily differentially expressed themselves, but might have a post-translational serovar-specific effect. Moreover, the small regulatory RNAs OmrA and OmrB connect differentially expressed proteins involved in carbohydrate metabolism.

### Differentially expressed proteins in *Salmonella* Typhimurium (STM) and Enteritidis (SENT) are associated with pathogenicity

**Fig 4:**
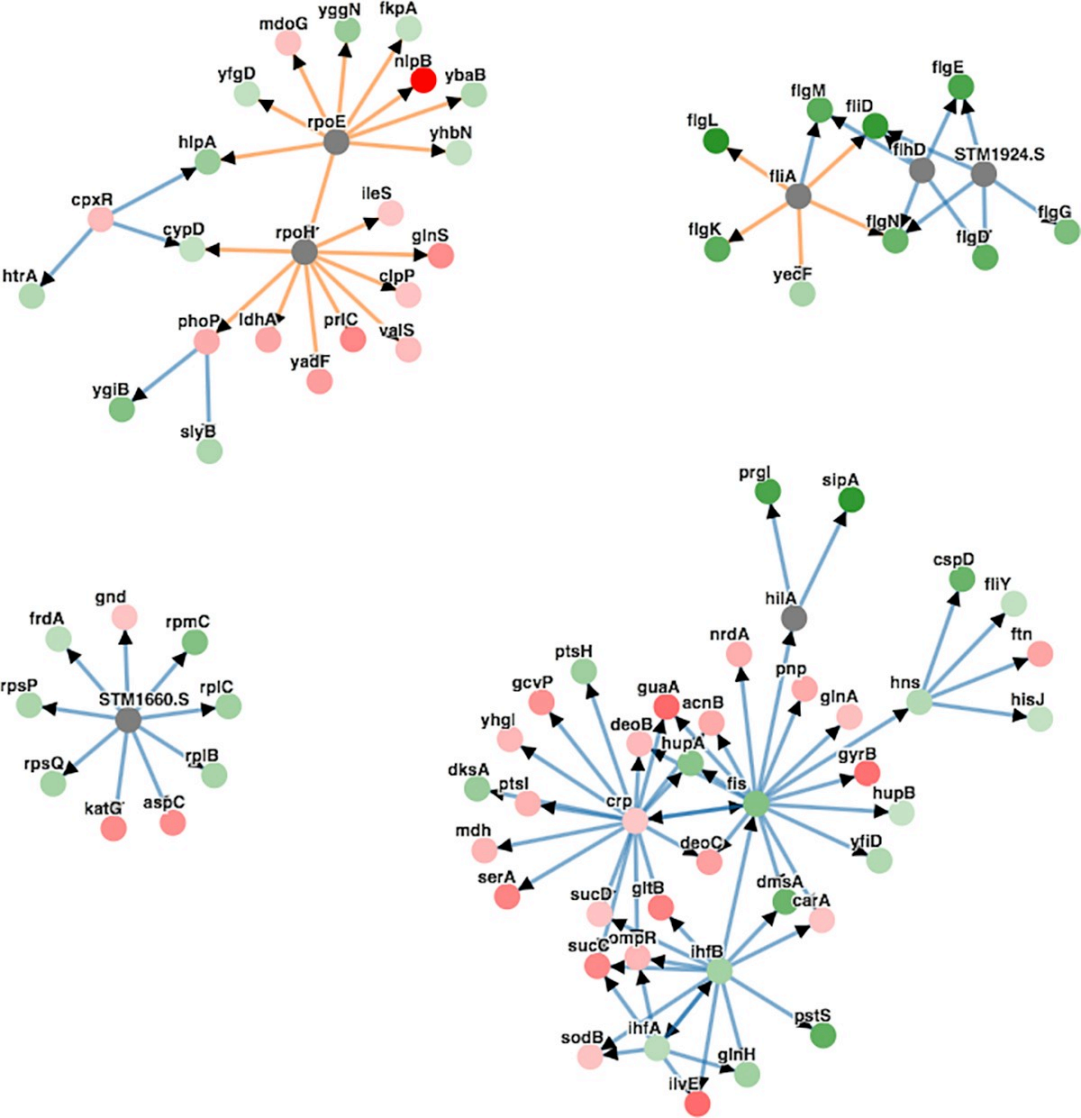
Phenetic sub-network inference analysis of differential protein expression in STM versus SENT. 78 out of 192 differentially expressed proteins are shown in the sub-network. Red nodes represent proteins with higher expression in SENT versus STM. Green nodes represent proteins with higher expression in STM versus SENT. The more intense the color, the higher the level of differential expression. Gray nodes have no differential expression. The color of the edge indicates the interaction type with blue referring to metabolic and orange to protein-DNA interactions.

A set of 465 core proteins were detected in all strains of STM and SENT. PCA of the LFQ intensities of these proteins showed a clear separation of the STM isolates from the SENT isolates based on the observed protein expression levels where the first two components cover ~80% of the total variation in expression levels (Fig 2C). The PCA also shows that the reference strains and the clinical isolates do not separate in STM and SENT. A total of 192 proteins with a minimal 2-fold change in their overall levels and an adjusted *P*-value lower than 0.05 were considered significantly differentially expressed between STM and SENT strains (S5 Table). GO enrichment analysis of these proteins showed that all GO terms with *P*<0.05 are related to pathogenesis (Table 2). The inferred subnetwork (Fig 4) revealed that the flagellar biosynthesis sigma factor FliA and the flagellar transcriptional regulators FlhD and FlhC (STM1924.S) connect the upregulated flagellar synthesis and motility proteins in STM. HilA, the main regulator of *Salmonella* Pathogenicity Island 1 (SPI-1), is possibly involved in the upregulation of the type 3 secretion system (T3SS) structural protein Prgl and effector protein SipA in STM.

## Discussion

The genomes of typhoidal and non-typhoidal *Salmonella* have a high level of similarity with more than 98% of sequence identity [32]. However, these two groups cause different diseases, host-pathogen interactions and immune responses. Here, we conducted the first comprehensive analysis of the proteomes of the *Salmonella* serovars Typhi, Paratyphi A, Typhimurium and Enteritidis using five clinical isolates that cover different geographical regions and one reference strain per *Salmonella* serovar. We have compared the expression levels of proteins from the core proteome under in vitro conditions and identified regulators that may help to explain the differences between different *Salmonella* serovars.

The classification of the four serovars into typhoidal and non-typhoidal groups is largely based on clinical presentation, with systemic and gastrointestinal disease, respectively. However, PCA of the LFQ intensities of the 418 detected proteins shared by all four serovars did not separate the typhoidal from the non-typhoidal serovars. Out of these 418 detected core proteins, 128 were significantly differentially expressed between typhoidal and the non-typhoidal serovars. However, GO analysis showed enrichment for proteins involved in translation and ribosomal activity, and thus largely represent the house keeping machinery of the bacterial cells. PCA showed that the LFQ intensities of the reference and clinical isolates within the STY, SPTA, STM and SENT serovars do not cluster separately, and the reference strains can thus be considered as representative for the serovar.

Further analysis showed that 230 proteins were differentially expressed between STY and SPTA. GO analysis revealed that proteins involved in carbohydrate and lipopolysaccharide metabolism, and proteins involved in external encapsulating structures were most enriched. The regulators in the sub-network analysis connecting the differentially expressed proteins are implicated in the cell envelope stress response and in polysaccharide metabolism. For example, OmrA/B connect Dld and SdaB, two proteins that are involved in transport of sugars and carbohydrate biosynthesis in *E*.*coli*, respectively. It is plausible that a serovar-specific effect acts at the sRNA-level, which is not detected in our proteomic analysis. CpxR that is known to have a role in the response to alterations in the cell envelope in *Salmonella* [33], explains the expression of Psd and LpxA required for phospholipid and glycolipid metabolism, respectively [34,35]. RpoS, RpoE and RpoH are involved in the stress response to different environmental conditions and contribute to *Salmonella* virulence [36–38]. CRP regulates the transcription of different operons involved in the transport of sugars and in catabolic functions [39], and FruR is required for carbohydrate metabolism [40]. The observation that cell surface proteins are significantly differently expressed between STY and SPTA is relevant for the diagnosis of *Salmonella* as well as for vaccination purposes. While the reference diagnostic method for typhoid fever is microbiological culture (blood, bone marrow or stool) and subsequent serotyping, rapid diagnostic tests (RDTs) have been developed and are commercially available for STY antigen and antibody detection [41]. However, diagnostic accuracy of the current RDTs is low, ranging from 31–97% [42] and more performant RDTs are urgently needed, including RDTs for SPTA. It has recently been shown that *Salmonella* antigen-based RDTs can be successfully applied to blood culture broths for *Salmonella* identification [43]. Three currently available typhoid vaccines are recommended by the WHO: an oral vaccine based on a live attenuated mutant strain of STY Ty21a (Ty21a), the injectable Vi capsular polysaccharide (ViCPS) vaccine and the typhoid conjugate vaccine (TCV) (http://www.who.int/immunization/policy/position_papers/typhoid/en/). However, these Typhi vaccines do not provide protection against paratyphoid fever caused by SPTA [44], and hence, a vaccine that protects against typhoid and paratyphoid fever would be of high value. When selecting antigens for developing new diagnostics or vaccines for both STY and SPTA, one should take into account that although encoded in both serovars, membrane proteins can be differentially expressed between both serovars and this should be tested in vitro and in vivo.

Upon comparing the proteomes of STM and SENT, 465 core proteins were detected, of which 192 were differentially expressed between the two serovars. GO enrichment analysis revealed that flagellar proteins and proteins involved in pathogenesis were most differentially expressed between both serovars. Among the higher expressed proteins in STM over SENT, six proteins are directly related to *Salmonella* pathogenicity island 1-encoded Type III secretion system (InvJ, SipA, SipD, SipC, PrgI, SipB). The T3SS-1 is an important virulence machinery that controls penetration of the gut epithelium during the infection by injecting effector proteins directly into the cytoplasm of epithelial cells through a needle-like appendages [45]. The regulator proteins InvJ and PrgI are known to be involved in needle and inner rod assembly [46], while SipA induces actin cytoskeletal rearrangements [47] and the translocases SipB and SipC form a translocation pore into the host cell membrane which is connected to the needle complex [48]. The sub-network also shows that HilA is possibly involved in the observed activation of the invasion proteins (SipA and PrgI) in STM. In addition, in the inferred sub-network the regulators FlhC (STM1924.S), FlhD and FliA were identified as regulators that connect 8 differentially expressed flagellar proteins (FlgL, FliD, FlgE, FlgM, FlgK, FlgD, FlgN, FlgG), showing higher expression profiles in Typhimurium strains. Besides their role in motility, flagellins were shown to stimulate both the innate and adaptive immune system and to cause inflammation upon STM infection [49]. Moreover, loss of flagellin expression in *Salmonella* has been linked to increased virulence in mice [50].

Some limitations in our study should be considered. The *Salmonella* strains were grown in standard *in vitro* conditions which may not be representative for protein expression in the infected host [51]. The addition of glucose to the medium may have induced catabolite repression. However, the addition of glucose as carbon source in needed to permit the growth of bacteria. Moreover, growth temperatures ranged between 35°C and 37°C and may have impacted expression levels. For instance, pathogenicity related gene expression is known to be temperature-sensitive [52]. In addition, the protein extraction procedure might have minorly affected the observed protein profiles although all steps have been performed on ice or 4°C. However, all strains have been grown using the same *in vitro* culture conditions and underwent the same extraction procedure and any possible effects are thus very likely averaged out in the comparative analysis. In addition, our mass spectrometry set-up is not as sensitive as the newest instruments currently available, and we captured around 20 to 40% of the proteomes. Poorly expressed proteins in the standard *in vitro* culture conditions used may thus have been missed, such as virulence related proteins [53]. Finally, the aim of our study was to conduct a comparative analysis of orthologous proteins shared between the four *Salmonella* serovars, and as such, we do not present information on serovar-specific (non-orthologous) proteins.

In conclusion, to the best of our knowledge this is the first study that compared the core proteomes of a large panel of clinical *Salmonella* isolates, covering the four clinically most relevant *Salmonella enterica* serovars: Typhi, Paratyphi A, Typhimurium and Enteritidis. Our comparative proteome analysis indicated differences in the expression of surface proteins between STY and SPTA, and in pathogenesis-related proteins between STM and SENT. Our insights may guide future developed of novel diagnostics and vaccines, and understanding of disease progression.

## Supporting information

S1 File Composition of Hi-Def Azure medium. (DOCX)

S2 File Principal component analysis (PCA) of LFQ intensities of expressed proteins within the same serovar. (DOCX)

S1 Table Protein concentration in μg/ml. (XLSX)

S2 Table Pearson correlation coefficients between two biological replicates. (XLSX)

S3 Table Differentially expressed proteins between non-typhoidal (NTS) and typhoidal Salmonella strains based on log2 fold change of LFQ intensity levels (log2 FC). (XLSX)

S4 Table Differentially expressed proteins between S. Paratyphi (SPTA) and S. Typhi (STY) based on log2 fold change of LFQ intensity levels (log2-FC). (XLSX)

S5 Table Differentially expressed proteins between between S. Enteritidis (SENT) and S. Typhimurium (STM) based on log2 fold change of LFQ intensity levels (log2-FC). (XLSX)

## References

[1] Crump JA, Mintz ED. Global trends in typhoid and paratyphoid fever. Clin Infect Dis. 2010 1 15;50(2):241–6. 10.1086/649541 20014951 PMC2798017

[2] Majowicz SE, Musto J, Scallan E, Angulo FJ, Kirk M, O’Brien SJ, et al The global burden of nontyphoidal *Salmonella* gastroenteritis. Clin Infect Dis. 2010 3 15;50(6):882–9. 10.1086/650733 20158401

[3] Buckle GC, Walker CLF, Black RE. Typhoid fever and paratyphoid fever: Systematic review to estimate global morbidity and mortality for 2010. J Glob Health. 2012;2: 010401 10.7189/jogh.02.010401 23198130 PMC3484760

[4] Crump JA, Luby SP, Mintz ED. The global burden of typhoid fever. Bull World Health Organ. 2004 5;82(5):346–53. 15298225 PMC2622843

[5] Bhan MK, Bahl R, Bhatnagar S. Typhoid and paratyphoid fever. Lancet. 2005 9 27;366(9487):749–62. 10.1016/S0140-6736(05)67181-4 16125594

[6] Ochiai RL, Wang X, von Seidlein L, Yang J, Bhutta ZA, Bhattacharya SK, et al *Salmonella* Paratyphi A Rates, Asia. Emerg Infect Dis. 2005 11;11(11):1764–6. 10.3201/eid1111.050168 16318734 PMC3367370

[7] Ao TT, Feasey NA, Gordon MA, Keddy KH, Angulo FJ, Crump JA. Global burden of invasive nontyphoidal Salmonella disease, 2010(1). Emerging Infect Dis. 2015 6;21(6). 10.3201/eid2106.140999 PMC4451910 25860298

[8] Feasey NA, Hadfield J, Keddy KH, Dallman TJ, Jacobs J, Deng X, et al Distinct *Salmonella* Enteritidis lineages associated with enterocolitis in high-income settings and invasive disease in low-income settings. Nat Genet. 2016;48(10):1211–7. 10.1038/ng.3644 27548315 PMC5047355

[9] Okoro CK, Kingsley RA, Connor TR, Harris SR, Parry CM, Al-Mashhadani MN, et al Intra-continental spread of human invasive *Salmonella* Typhimurium pathovariants in sub-Saharan Africa. Nat Genet. 2012 11;44(11):1215–21. 10.1038/ng.2423 23023330 PMC3491877

[10] Zou Q-H, Li R-Q, Liu G-R, Liu S-L. Comparative genomic analysis between typhoidal and non-typhoidal *Salmonella* serovars reveals typhoid-specific protein families. Infect Genet Evol. 2014 8;26:295–302. 10.1016/j.meegid.2014.06.008 24951835

[11] Nuccio S-P, Bäumler AJ. Comparative Analysis of Salmonella Genomes Identifies a Metabolic Network for Escalating Growth in the Inflamed Gut. mBio. 2014 5 1;5(2):e00929–14. 10.1128/mBio.00929-14 24643865 PMC3967523

[12] Yang L, Tan J, O’Brien EJ, Monk JM, Kim D, Li HJ, et al Systems biology definition of the core proteome of metabolism and expression is consistent with high-throughput data. Proc Natl Acad Sci USA. 2015 8 25;112(34):10810–5. 10.1073/pnas.1501384112 26261351 PMC4553782

[13] Idelevich EA, Schüle I, Grünastel B, Wüllenweber J, Peters G, Becker K. Rapid identification of microorganisms from positive blood cultures by MALDI-TOF mass spectrometry subsequent to very short-term incubation on solid medium. Clin Microbiol Infect. 2014 10;20(10):1001–6. 10.1111/1469-0691.12640 24698361

[14] Brown RN, Sanford JA, Park JH, Deatherage BL, Champion BL, Smith RD, et al A Comprehensive Subcellular Proteomic Survey of *Salmonella* Grown under Phagosome-Mimicking versus Standard Laboratory Conditions. Int J Proteomics. 2012;2012:123076 10.1155/2012/123076 22900174 PMC3410353

[15] Kim K, Yang E, Vu G-P, Gong H, Su J, Liu F, et al Mass spectrometry-based quantitative proteomic analysis of *Salmonella enterica* serovar Enteritidis protein expression upon exposure to hydrogen peroxide. BMC Microbiology. 2010 6 8;10:166 10.1186/1471-2180-10-166 20529336 PMC2897801

[16] Shi L, Adkins JN, Coleman JR, Schepmoes AA, Dohnkova A, Mottaz HM, et al Proteomic analysis of *Salmonella enterica* serovar typhimurium isolated from RAW 264.7 macrophages: identification of a novel protein that contributes to the replication of serovar typhimurium inside macrophages. J Biol Chem. 2006 9 29;281(39):29131–40. 10.1074/jbc.M604640200 16893888

[17] Coldham NG, Randall LP, Piddock LJV, Woodward MJ. Effect of fluoroquinolone exposure on the proteome of *Salmonella enterica* serovar Typhimurium. J Antimicrob Chemother. 2006 12;58(6):1145–53. 10.1093/jac/dkl413 17062612

[18] Niemann GS, Brown RN, Gustin JK, Stufkens A, Shaikh-Kidwai AS, Li J, et al Discovery of novel secreted virulence factors from *Salmonella enterica* serovar Typhimurium by proteomic analysis of culture supernatants. Infect Immun. 2011 1;79(1):33–43. 10.1128/IAI.00771-10 20974834 PMC3019877

[19] Webber MA, Coldham NG, Woodward MJ, Piddock LJV. Proteomic analysis of triclosan resistance in *Salmonella enterica* serovar Typhimurium. J Antimicrob Chemother. 2008 7;62(1):92–7. 10.1093/jac/dkn138 18388111

[20] Charles RC, Harris JB, Chase MR, Lebrun LM, Sheikh A, LaRocque RC, et al Comparative proteomic analysis of the PhoP regulon in Salmonella enterica serovar Typhi versus Typhimurium. PLoS ONE. 2009 9 10;4(9):e6994 10.1371/journal.pone.0006994 19746165 PMC2736619

[21] Feng Y, Chien K-Y, Chen H-L, Chiu C-H. Pseudogene recoding revealed from proteomic analysis of *Salmonella* serovars. J Proteome Res. 2012 3 2;11(3):1715–9. 10.1021/pr200904c 22296100

[22] Wang Y, Huang K-Y, Huo Y. Proteomic comparison between *Salmonella* Typhimurium and Salmonella Typhi. J Microbiol. 2014 1;52(1):71–6. 10.1007/s12275-014-3204-3 24390840

[23] Neidhardt FC, Bloch PL, Smith DF. Culture Medium for Enterobacteria. J Bacteriol. 1974 9;119(3):736–47. 4604283 10.1128/jb.119.3.736-747.1974 PMC245675

[24] Cox J, Mann M. MaxQuant enables high peptide identification rates, individualized p.p.b.-range mass accuracies and proteome-wide protein quantification. Nat Biotech. 2008 12;26(12):1367–72. 10.1038/nbt.1511 19029910

[25] Parkhill J, Dougan G, James KD, Thomson NR, Pickard D, Wain J, et al Complete genome sequence of a multiple drug resistant *Salmonella enterica* serovar Typhi CT18. Nature. 2001 10 25;413(6858):848–52. 10.1038/35101607 11677608

[26] McClelland M, Sanderson KE, Clifton SW, Latreille P, Porwollik S, Sabo A, et al Comparison of genome degradation in Paratyphi A and Typhi, human-restricted serovars of *Salmonella enterica* that cause typhoid. Nat Genet. 2004 12;36(12):1268–74. 10.1038/ng1470 15531882

[27] Jarvik T, Smillie C, Groisman EA, Ochman H. Short-Term Signatures of Evolutionary Change in the *Salmonella enterica* Serovar Typhimurium 14028 Genome. J Bacteriol. 2010 1;192(2):560–7. 10.1128/JB.01233-09 19897643 PMC2805332

[28] Thomson NR, Clayton DJ, Windhorst D, Vernikos G, Davidson S, Churcher C, et al Comparative genome analysis of *Salmonella* Enteritidis PT4 and *Salmonella* Gallinarum 287/91 provides insights into evolutionary and host adaptation pathways. Genome Res. 2008 10;18(10):1624–37. 10.1101/gr.077404.108 18583645 PMC2556274

[29] Altenhoff AM, Škunca N, Glover N, Train C-M, Sueki A, Piližota I, et al The OMA orthology database in 2015: function predictions, better plant support, synteny view and other improvements. Nucleic Acids Res. 2015 1;43(Database issue):D240–249. 10.1093/nar/gku1158 25399418 PMC4383958

[30] Huang DW, Sherman BT, Lempicki RA. Systematic and integrative analysis of large gene lists using DAVID bioinformatics resources. Nat Protoc. 2009;4(1):44–57. 10.1038/nprot.2008.211 19131956

[31] De Maeyer D, Weytjens B, Renkens J, De Raedt L, Marchal K. PheNetic: network-based interpretation of molecular profiling data. Nucl Acids Res. 2015 4 15;gkv347. 10.1093/nar/gkv347 PMC4489255 25878035

[32] McClelland M, Sanderson KE, Spieth J, Clifton SW, Latreille P, Courtney L, et al Complete genome sequence of *Salmonella enterica* serovar Typhimurium LT2. Nature. 2001 10 25;413(6858):852–6. 10.1038/35101614 11677609

[33] Nandre RM, Mahajan P. Molecular Significance of lon and cpxR Genes in the Pathogenicity of *Salmonella*. Open Journal of Animal Sciences. 2015 9 23;05(04):429.

[34] Dowhan W. A retrospective: Use of *Escherichia coli* as a vehicle to study phospholipid synthesis and function. Biochim Biophys Acta. 2013 3;1831(3):471–94. 10.1016/j.bbalip.2012.08.007 22925633 PMC3513495

[35] Zhou P, Zhao J. Structure, Inhibition, and Regulation of Essential Lipid A Enzymes. Biochim Biophys Acta. 2017 11;1862(11):1424–38. 10.1016/j.bbalip.2016.11.014 PMC5466501 27940308

[36] Bang I-S, Frye JG, McClelland M, Velayudhan J, Fang FC. Alternative sigma factor interactions in *Salmonella*: sigma and sigma promote antioxidant defences by enhancing sigma levels. Mol Microbiol. 2005 5;56(3):811–23. 10.1111/j.1365-2958.2005.04580.x 15819634

[37] Cho Y, Park YM, Barate AK, Park S-Y, Park HJ, Lee MR, et al The role of rpoS, hmp, and ssrAB in *Salmonella enterica* Gallinarum and evaluation of a triple-deletion mutant as a live vaccine candidate in Lohmann layer chickens. Journal of Veterinary Science. 2015 6;16(2):187 10.4142/jvs.2015.16.2.187 25549217 PMC4483502

[38] Kazmierczak MJ, Wiedmann M, Boor KJ. Alternative Sigma Factors and Their Roles in Bacterial Virulence. Microbiol Mol Biol Rev. 2005 12;69(4):527–43. 10.1128/MMBR.69.4.527-543.2005 16339734 PMC1306804

[39] Botsford JL, Harman JG. Cyclic AMP in prokaryotes. Microbiol Rev. 1992 3;56(1):100–22. 1315922 10.1128/mr.56.1.100-122.1992 PMC372856

[40] Vartak NB, Reizer J, Reizer A, Gripp JT, Groisman EA, Wu LF, et al Sequence and evolution of the FruR protein of *Salmonella* typhimurium: a pleiotropic transcriptional regulatory protein possessing both activator and repressor functions which is homologous to the periplasmic ribose-binding protein. Res Microbiol. 1991 12;142(9):951–63. 1805309 10.1016/0923-2508(91)90005-u

[41] Rahman M, Siddique AK, Tam FC-H, Sharmin S, Rashid H, Iqbal A, et al Rapid detection of early typhoid fever in endemic community children by the TUBEX O9-antibody test. Diagnostic Microbiology and Infectious Disease. 2007 7 1;58(3):275–81. 10.1016/j.diagmicrobio.2007.01.010 17350203

[42] Thriemer K, Ley B, Menten J, Jacobs J, van den Ende J. A systematic review and meta-analysis of the performance of two point of care typhoid fever tests, Tubex TF and Typhidot, in endemic countries. PLoS ONE. 2013;8(12):e81263 10.1371/journal.pone.0081263 24358109 PMC3864786

[43] Kuijpers LMF, Chung P, Peeters M, Phoba M-F, Kham C, Barbé B, et al Diagnostic accuracy of antigen-based immunochromatographic rapid diagnostic tests for the detection of *Salmonella* in blood culture broth. PLoS One 13(3): e0194024 10.1371/journal.pone.0194024 29518166 PMC5843332

[44] Mahon BE, Newton AE, Mintz ED. Effectiveness of typhoid vaccination in US travelers. Vaccine. 2014 6 17;32(29):3577–9. 10.1016/j.vaccine.2014.04.055 24837780 PMC4604742

[45] Galán JE, Wolf-Watz H. Protein delivery into eukaryotic cells by type III secretion machines. Nature. 2006 11 30;444(7119):567–73. 10.1038/nature05272 17136086

[46] Monjarás Feria JV, Lefebre MD, Stierhof Y-D, Galán JE, Wagner S. Role of autocleavage in the function of a type III secretion specificity switch protein in Salmonella enterica serovar Typhimurium. MBio. 2015 10 13;6(5):e01459–01415. 10.1128/mBio.01459-15 26463164 PMC4620466

[47] Patel JC, Galán JE. Manipulation of the host actin cytoskeleton by *Salmonella*—all in the name of entry. Curr Opin Microbiol. 2005 2;8(1):10–5. 10.1016/j.mib.2004.09.001 15694851

[48] Kim JS, Eom JS, Jang JI, Kim HG, Seo DW, Bang I-S, et al Role of Salmonella Pathogenicity Island 1 protein IacP in *Salmonella enterica* serovar typhimurium pathogenesis. Infect Immun. 2011 4;79(4):1440–50. 10.1128/IAI.01231-10 21263021 PMC3067539

[49] Olsen JE, Hoegh-Andersen KH, Casadesús J, Rosenkranzt J, Chadfield MS, Thomsen LE. The role of flagella and chemotaxis genes in host pathogen interaction of the host adapted Salmonella enterica serovar Dublin compared to the broad host range serovar S. Typhimurium. BMC Microbiol. 2013 3 25;13:67 10.1186/1471-2180-13-67 23530934 PMC3621167

[50] Ikeda JS, Schmitt CK, Darnell SC, Watson PR, Bispham J, Wallis TS, et al Flagellar Phase Variation of *Salmonella enterica* Serovar Typhimurium Contributes to Virulence in the Murine Typhoid Infection Model but Does Not Influence *Salmonella*-Induced Enteropathogenesis. Infect Immun. 2001 5;69(5):3021–30. 10.1128/IAI.69.5.3021-3030.2001 11292720 PMC98256

[51] Liu Y, Zhang Q, Hu M, Yu K, Fu J, Zhou F, et al Proteomic Analyses of Intracellular *Salmonella enterica* Serovar Typhimurium Reveal Extensive Bacterial Adaptations to Infected Host Epithelial Cells. Infect Immun. 2015 7;83(7):2897–906. 10.1128/IAI.02882-14 25939512 PMC4468536

[52] Lam O, Wheeler J, Tang CM. Thermal control of virulence factors in bacteria: A hot topic. Virulence. 2014 12 10;5(8):852–62. 10.4161/21505594.2014.970949 25494856 PMC4601195

[53] Espadas G, Borràs E, Chiva C, Sabidó E. Evaluation of different peptide fragmentation types and mass analyzers in data-dependent methods using an Orbitrap Fusion Lumos Tribrid mass spectrometer. Proteomics. 2017 5;17(9). 10.1002/pmic.201600416 28266123

