# Supplementary material for: Salmonella Typhi, Paratyphi A, Enteritidis and Typhimurium core proteomes reveal differentially expressed proteins linked to the cell surface and pathogenicity
Source: PLoS Negl Trop Dis. 2019 May 24;13(5):e0007416. doi: 10.1371/journal.pntd.0007416 (PMC6553789; doi:10.1371/journal.pntd.0007416)
Supplement: S1 File — (DOCX) [file pntd.0007416.s001.docx]

**S1 File: Composition of the Hi-Def Azure Medium.**

| L-Alanine |
| --- |
| L-Arginine |
| L-Asparagine |
| L-Aspartic Acid |
| L-Glutamic Acid |
| L-Glutamine |
| Glycine |
| L-Histidine |
| L-Isoleucine |
| L-Proline |
| L-Serine |
| L-Threonine |
| L-Tryptophan |
| L-Valine |
| Leucine |
| Lysine |
| Methionine |
| Phenylalanine |
| Cysteine |
| Tyrosine |
| Potassium Phosphate |
| MOPS |
| Tricine |
| Iron Sulfate |
| Ammonium Chloride |
| Potassium Sulfate |
| Calcium Chloride |
| Magnesium Chloride |
| Sodium Chloride |
| Ammonium Molybdate |
| Boric Acid |
| Cobalt Chloride |
| Cupric Sulfate |
| Manganese Chloride |
| Zinc Sulfate |
| Adenine |
| Cytosine |
| Manganese Chloride |
| Zinc Sulfate |
| Uracil |
| Guanine |
| Thiamine |
| Calcium Pantothenate |
| para-Amino Benzoic Acid |
| para-Hydroxy Benzoic Acid |
| 2,3-di Hydroxy Benzoic Acid |
