# Supplementary material for: Salmonella Typhi, Paratyphi A, Enteritidis and Typhimurium core proteomes reveal differentially expressed proteins linked to the cell surface and pathogenicity
Source: PLoS Negl Trop Dis. 2019 May 24;13(5):e0007416. doi: 10.1371/journal.pntd.0007416 (PMC6553789; doi:10.1371/journal.pntd.0007416)
Supplement: S2 File — (DOCX) [file pntd.0007416.s002.docx]

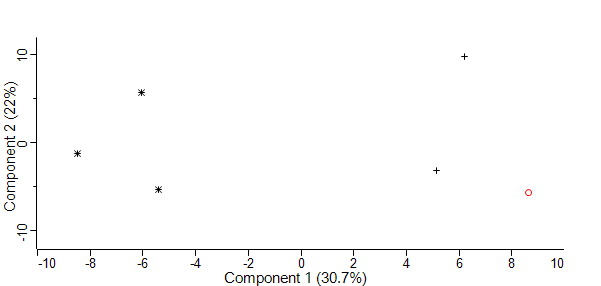


**(A)**

**(B)**


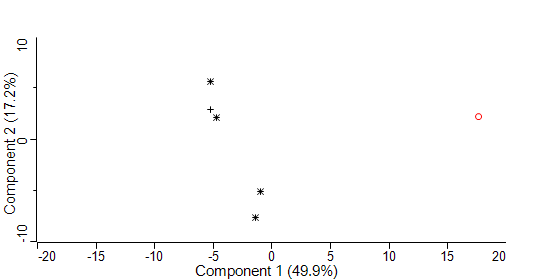


**(C)**


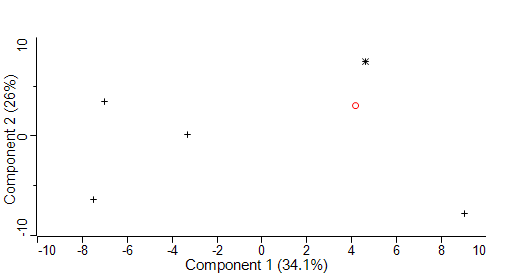


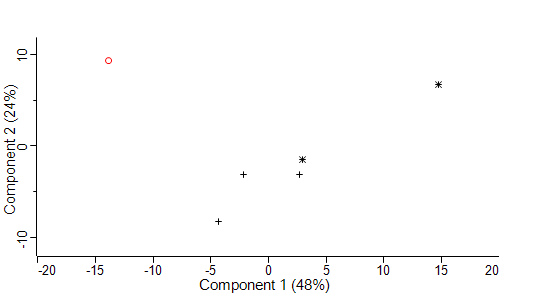


**(D)**

**S2 File. Principal Component Analysis (PCA) of LFQ intensities of expressed proteins within the**

**same serovar.** The PCA plots show the first and second principle components among the STY

strains **(A)**, SPTA strains **(B)**, STM strains **(C)** and SENT strains **(D)**. Reference strains for each serovar

are presented in red. * represent Asian strains and + represent African strains.
